# Impact of an Interdisciplinary Educational Intervention on Healthcare Provider Knowledge and Beliefs Regarding Opioid Harm Reduction in Older Adults: A Pre-Post Survey Study

**DOI:** 10.3390/pharmacy14030086

**Published:** 2026-06-16

**Authors:** Ariel Dulaney, Anne Taylor, Haley Phillippe, Renee Delaney, Lindsey Hohmann

**Affiliations:** Harrison College of Pharmacy, Auburn University, 2316 Walker Building, Auburn, AL 36849, USA

**Keywords:** opioids, misuse, older adults, elderly

## Abstract

Opioid misuse continues to be a major public health issue in the United States. Older adults (≥65) are at particular risk of harm from opioids due to changes in opioid pharmacokinetics with age; however, healthcare professionals lack training and confidence in addressing opioid harm reduction strategies in this population. Therefore, the purpose of this study was to improve healthcare professional knowledge and beliefs regarding opioid harm reduction strategies amongst older adults. An 8 h interprofessional conference was conducted 1 May 2025 to educate healthcare providers about opioid misuse prevention strategies for older adults. This study utilized a quasi-experimental one-group pretest–posttest design to assess changes in healthcare professional knowledge and beliefs before and after the conference. Healthcare professionals in the U.S. were recruited to participate in the conference via email listservs with national reach, predominantly concentrated in Alabama. Data were collected at pre- and post-conference via an anonymous online survey informed by the Theory of Planned Behavior and Health Belief Model. Primary outcome measures included: (1) knowledge of opioid use and misuse in older adults (5 items); (2) prescribing and dispensing attitudes surrounding opioids and medications for opioid use disorder (MOUD) (5 items); (3) perceived susceptibility to harm from opioids (4 items); and (4) perceived barriers to opioid harm reduction in older adults (17-items). Constructs were measured using multiple-choice questions (knowledge) and Likert-type scales (1 = strongly disagree, 5 = strongly agree). Secondarily, intention to join a Microsoft Teams working group for ongoing collaboration was assessed through a single categorical (Yes/No/Unsure) multiple-choice question at post-conference. Data were analyzed using descriptive statistics, and differences in mean knowledge, attitudes, susceptibility, and barriers scale scores from pre- to post-conference were analyzed using Wilcoxon signed-rank tests (alpha = 0.05). Of N = 75 survey respondents, the majority were White (86.7%), female (74.7%), 50 years of age on average, and employed as pharmacists (68%). Overall, mean (SD) knowledge (83.73% [19.92] versus 90.67% [12.45]; *p* = 0.011) and perceived susceptibility (3.82 [0.63] versus 4.03 [0.63]; *p* = 0.002) increased from pre- to post-conference, while perceived barriers decreased (2.71 [0.54] versus 2.54 [0.58]; *p* = 0.001). Despite an upward trend, there was no statistically significant change in the mean prescribing and dispensing attitudes from baseline to post-conference. Additionally, 34.7% intended to join the Microsoft Teams working group at post-conference. Findings support the utility of interprofessional educational interventions to increase healthcare provider knowledge and beliefs regarding opioid harm reduction strategies amongst older adults.

## 1. Introduction

Opioid misuse continues to be a major public health issue in the United States. Over 120 million prescriptions for opioid pain relievers were dispensed in 2024 [1], and 54,045 people died due to opioid overdose [2]. In the Deep South, the opioid prescribing rate is disproportionately higher, with the state of Alabama having one of the highest opioid dispensing rates in the nation at 68.5 per 100 persons versus 35.4 nationwide [3]. Furthermore, dispensing and overdose rates differ in counties within Alabama [3], with only 216 substance use disorder treatment facilities statewide [4]. Similarly limited resources and healthcare personnel are echoed in other Deep South States [4,5,6]. This landscape of scarce resources leads to disparities and gaps in care, with over 80% of people failing to receive needed treatment for opioid use disorder (OUD) [7], and inordinately affects vulnerable populations who are at increased risk of harm from opioids, underserved, and/or disproportionately affected by the opioid crisis.

Older adults (≥65 years) are one such vulnerable population who are at increased risk of harm from opioids [8,9,10,11]. Changes in opioid pharmacokinetics with age lead to increased likelihood of negative side effects from opioids, including respiratory depression, sedation, and overdose [8]. Approximately 10% of older adults progress to chronic (>3 months) opioid use post-surgery [9], with odds of developing OUD exacerbated in older adults experiencing social isolation [10], a not uncommon phenomenon in the rural Deep South. Although rates of fatal opioid overdose are lower among older adults (7.0 per 100,000) [12] versus all ages (16.0 per 100,000) [13], rates increased 63% from 2019 to 2024 [12], making this a priority population for intervention. Thus, it is crucial that healthcare providers address potential opioid misuse and harm reduction strategies when treating older adult patients. However, older adults remain underrepresented in OUD screening, treatment protocols, and harm reduction strategies [12,14]. Clinical guidelines are often geared toward younger populations, and age-related stigma can contribute to both underdiagnosis and inadequate care [14]. Importantly, signs of substance misuse in older patients may be misattributed to cognitive decline, frailty, or the normal aging process, leading to missed opportunities for intervention [14,15]. Without increased provider awareness, this trend is likely to continue upward, further straining healthcare systems and diminishing quality of life for aging populations.

Healthcare professionals play distinct but complementary roles in opioid harm reduction [16,17]. Prescribers (e.g., physicians, nurse practitioners) are primarily responsible for initiating and managing pharmacologic therapy, while pharmacists and other professionals contribute through medication management, patient education, and harm reduction interventions such as naloxone dispensing [17,18]. While prescribing authority is typically associated with physicians and certain advanced practice providers, pharmacists may also engage in prescribing activities under collaborative practice agreements or state-specific authority, including naloxone and, in some settings, buprenorphine [19,20,21,22]. Given this variability, the importance of interdisciplinary education supporting the spectrum of opioid safety and harm reduction roles within and across professions is critical [16,23]. Indeed, interprofessional education (IPE) has been identified as an effective strategy to enhance collaborative practice, improve communication across disciplines, and improve patient outcomes [23]. Therefore, the purpose of this study was to improve healthcare professional knowledge and beliefs regarding opioid harm reduction strategies amongst older adults via an interprofessional educational intervention. Ultimately, this may serve to expand the capacity for harm reduction efforts among older adults.

## 2. Materials and Methods

### 2.1. Study Design

An 8 h interprofessional conference was conducted on 1 May 2025 to educate healthcare providers in Alabama and nationally about opioid misuse prevention strategies for the older adult population. The conference was conducted by the Auburn University Harrison College of Pharmacy (HCOP) and funded by the Agency for Healthcare Research and Quality (AHRQ; grant number 1R13HS029600-01A1). This study utilized a quasi-experimental one-group pretest–posttest design to assess changes in participant knowledge and beliefs before and after the conference. All study procedures were reviewed and approved by the Institutional Review Board (IRB) at the corresponding author’s institution (Protocol #24-662 EX 2407).

### 2.2. Participants and Recruitment

Healthcare professionals (behavioral/mental health specialists, physicians, physician assistants, nurses, nurse practitioners, dentists, dental hygienists, pharmacists, pharmacy technicians, social workers) ≥ 18 years of age across the U.S. were eligible to participate. Individuals were recruited to participate in the conference using purposive sampling via electronic newsletters distributed through two healthcare provider email listservs: the HCOP Center for Opioid Research, Education, and Outreach (COACH) (approximately 3100 subscribers) and the HCOP Office of Continuing Education (approximately 5151 subscribers). These listservs include a range of healthcare professionals, encompassing behavioral/mental health specialists, physicians, physician assistants, nurses, nurse practitioners, dentists, dental hygienists, pharmacists, pharmacy technicians, and social workers. While the majority of subscribers are located in Alabama, both listservs include members from all U.S. states. A target of approximately 80–100 conference participants was determined based on in-person conference venue capacity. Recruitment began 4 months prior to the conference with an initial “save-the-date” email invitation, with a total of 4 email reminders to register for the conference. Participants received 8 h of continuing professional education credit for attending the conference.

### 2.3. Sample Size Estimation

A power calculation was conducted using G*Power software version 3.1.9.7 (Heinrich-Heine-Universität Düsseldorf, Düsseldorf, Germany) [24,25]. Assuming a small effect size (dz = 0.4) [26], alpha of 0.05, and power of 0.80, a minimum sample size of 60 was determined to be sufficient to assess differences in primary outcome measures (average knowledge and beliefs) from pre- to post-conference.

### 2.4. Conference Development and Implementation

An 8 h interprofessional conference, entitled “2025 COACH Annual Conference: Disparities in Opioid Harm Reduction in Older Adults,” was developed by the authors (LH, HP, RD, AT) in consultation with a stakeholder panel. The stakeholder panel consisted of lived experience experts (*n* = 2), both of whom: (1) were in recovery from substance misuse; and (2) worked with older adults with substance use disorders. Panelists were recruited via email from amongst authors’ contacts from previous work in Alabama. Three one-hour virtual meetings were conducted with the panelists prior to the conference, including two initial meetings between the authors and each panelist to orient everyone to the project, and a final meeting to review programming. During meetings and via interim email communications, the panelists provided feedback on conference content and format.

The conference was held on 1 May 2025. To maximize participation, a hybrid conference attendance model was utilized, including an in-person option on the HCOP campus for local Alabama residents, and a live virtual option via Zoom version 5.17.5 (Zoom Communications, Inc., San Jose, CA, USA) to reach a nationwide audience. Of note, although continuing education credit required participation in the full conference, full-duration attendance for virtual participants could not feasibly be verified. Conference components consisted of: (1) didactic lectures; (2) a lived experience panel; and (3) a Microsoft Teams COACH Annual Conference working group.

#### 2.4.1. Didactic Lectures

Didactic programming was delivered via traditional slideshow presentations, utilizing expert speakers recruited via email from amongst: pharmacists and researchers employed at HCOP; and pharmacists, physicians, and mental health counselors practicing in the local Alabama community. There were a total of 9 didactic sessions (Table 1), each ranging from 25–60 min in duration, including: (1) Current Overdose Trends in the United States and in Older Adults; (2) Opioid Prescribing in the Elderly; (3) Introduction to Medications for Treatment of Opioid Use Disorder; (4) Substance Use Resources for Older Populations; (5) Managing Opioid Overdose and Naloxone; (6) Prescription Drug Monitoring Program (PDMP); (7) Drugs of Abuse and Fentanyl Test Strips; (8) Medication Labeling Best Practices Among Older Adults; and (9) Enhancing Patient Care Through Collaboration. Each session emphasized key issues of concern for the older adult population.

#### 2.4.2. Lived Experience Panel

A 50 min lived experience panel supplemented the didactic content. The panel was moderated by one of the authors (HP), with the two stakeholder panelists serving as speakers. This session utilized a conversational discussion-style format with a list of pre-arranged questions posed by the moderator for each panelist to respond. Questions were developed in collaboration between the authors and panelists, and focused on highlighting real-life issues faced by individuals with substance use disorder, including particular areas of concern encountered when treating older adult patients (e.g., communication style, prescription sharing).

#### 2.4.3. Microsoft Teams Working Group

A Microsoft Teams COACH Annual Conference working group was created by the authors to allow conference attendees to communicate and collaborate with each other post-conference regarding issues related to substance misuse and older adults. Several days prior to the conference, an email invitation was sent to all registered conference attendees via the Teams page. In order to join the working group, attendees had to accept the invitation to the Teams page. After accepting the invitation, attendees were able to view or post comments and files within any of the four channels: (1) general; (2) community members; (3) healthcare professionals; and (4) law enforcement and first responders. These channels were created to allow a place for more targeted conversations and information to be housed. Of note, the Teams invitation was distributed only to conference registrants; however, attendance verification for all individuals who joined the Teams platform could not be confirmed. Post-conference, the authors continue to post relevant and timely information related to each channel (e.g., future healthcare provider conferences, Medicare Open Enrollment dates for older adult patients).

### 2.5. Data Collection and Measures

Data were collected via an anonymous online survey at pre-conference (baseline) and post-conference via the Qualtrics^®^ survey platform (Qualtrics, Provo, UT, USA). Surveys were distributed via QR codes at the beginning and end of the conference, with time built into the conference to allow for survey completion. To maintain anonymity, a unique code was assigned to each participant on the baseline survey; participants entered this code on the post-conference survey to allow for matching of individual pre- and post-responses.

The pre- and post-survey instruments consisted of 4 primary outcome measures: (1) knowledge of opioid use and misuse in older adults (5-items); (2) prescribing and dispensing attitudes surrounding opioids and medications for opioid use disorder (MOUD) (5-items); (3) perceived susceptibility to harm from opioids (4-items); and (4) perceived barriers to opioid harm reduction in older adults (17-items). Knowledge was measured via multiple-choice questions, while beliefs (attitudes, perceived susceptibility, and perceived barriers) were measured using 5-point Likert-type scales (1 = strongly disagree to 5 = strongly agree).

Secondarily, the baseline survey also assessed participant demographics including profession, sex, race and ethnicity, age, and geographic location (state and zip code) via multiple-choice and free-response questions. Rural-Urban Commuting Area (RUCA) codes were used to determine rural or urban residence (urban: RUCA codes 1–3; rural: RUCA codes 4–10) based on zip code [27]. Furthermore, intention to join the Microsoft Teams COACH Annual Conference working group was assessed on the post-survey through a single categorical (Yes/No/Unsure) multiple-choice question.

The baseline and post-conference survey instruments were developed by the investigators, with measures and items informed by theoretical frameworks [28,29,30] and published literature [7,8,9,10,31,32]. Specifically, attitude and intention constructs were informed by the Theory of Planned Behavior [28], and the perceived susceptibility and perceived barriers scales were guided by the Health Belief Model [29,30]. Knowledge items were informed by the Centers for Disease Control and Prevention (CDC) Clinical Practice Guideline for Prescribing Opioids for Pain [31] and the American Geriatrics Society (AGS) Beers Criteria [32], as well as work published by Krawczyk [7], Wilder-Smith [8], Santosa [9], and Yang [10] and colleagues. Expert speakers delivering each session were also consulted to ensure that knowledge questions aligned with their content. Additionally, according to best practices in interprofessional education [16], attitude and perception items referring to “prescribe/dispense” were intentionally phrased to reflect medication-related responsibilities across professions; respondents were expected to interpret these items according to their individual scope of practice and role (e.g., prescribing, dispensing, or both). Survey instruments were pre-tested for face and content validity among topic matter experts at the investigators’ institution (*n* = 4), and items were modified based on feedback prior to distribution. The full survey instruments are available in Supplemental File S1.

### 2.6. Data Analysis

Participant demographics and outcome measures were characterized using descriptive statistics (frequencies, percentages, means, standard deviations). The number of knowledge questions answered correctly was averaged across the sample to calculate an overall mean knowledge scale score (percent correct). Attitudes, perceived susceptibility, and perceived barriers Likert-type scale items were summed and averaged to create total mean scale scores, with higher mean values indicating more of a construct (e.g., greater willingness to prescribe, higher perceived susceptibility, more perceived barriers). Missing data due to item non-response was dropped from analysis. Internal consistency of belief scales was assessed via the Cronbach’s alpha statistic. Differences in mean scale scores from pre- to post-conference were analyzed using two-sided Wilcoxon signed-rank tests (data were non-parametric as indicated by Komogorov-Smirnov *p* < 0.05), with effect size estimates (*r* = |Z|/√N) calculated and interpreted (*r* = 0.1 small effect, *r* = 0.3 medium effect, *r* ≥ 0.5 large effect) based on Pautz and colleagues’ recommendations for Wilcoxon signed-rank tests [33]. Changes in the proportion of individuals who answered each knowledge item correctly (dichotomized as correct versus incorrect) from pre- to post-conference were analyzed using two-sided McNemar’s tests, with odds ratios (ORs) utilized as estimates of effect size (OR = 1.44 small effect, OR = 2.48 medium effect, OR ≥ 4.27 large effect) [34]. Reporting was guided by the Transparent Reporting of Evaluations with Nonrandomized Designs (TREND) checklist (Supplemental File S2) [35].

## 3. Results

### 3.1. Participant Characteristics

A total of 187 healthcare professionals attended the conference, with the majority of individuals attending virtually (*n* = 154, 82.35%). Among all attendees, 106 baseline surveys (response rate = 56.68%) and 93 post-conference surveys (response rate = 49.73%) were submitted, with 75 individuals completing surveys at both timepoints (useable response rate = 40.11%). Of these 75 survey respondents, the highest attendance was from pharmacists (68%), followed by nurses and nurse practitioners (10.7%), social workers and behavioral/mental health specialists (10.7%), pharmacy technicians (4.0%), and physicians (2.7%) (Table 2). The majority of participants were White (86.7%), female (74.7%), with an average age of 50 years. Residents of nine different US states attended, with the largest proportions of attendees located in urban regions (84.9%) and in the Deep South states of Alabama (78.4%), Georgia (5.4%), and Mississippi (4.1%).

### 3.2. Knowledge

Overall, the mean (SD) knowledge score increased from pre- to post-conference (83.73% [19.92] versus 90.67% [12.45]; *r* = 0.295, *p* = 0.011) (Table 3). Specifically, 64% of participants at pre-conference versus 78.4% at post-conference correctly identified that only 20% of people receive needed treatment for opioid use disorder (OUD) (*p* = 0.054). Other items scored high at baseline with small, statistically non-significant knowledge increases from pre- to post-conference, including: changes in opioid pharmacokinetics with age lead to increased likelihood of negative side effects from opioids (93.3 to 97.3%); about 10% of older adults progress to chronic (>3 months) opioid use after surgery (94.7 to 97.3%); and the odds of developing OUD are exacerbated in older adults experiencing social isolation (81.2 to 82.7%). These high baseline knowledge scores suggest a potential ceiling effect, limiting the magnitude of observable improvement. Participants likewise showed small but statistically significant pre–post increases in knowledge regarding increased risk of respiratory depression (94.7 to 100.0%), sedation (92.0 to 97.3%), or overdose (88.0 to 96.0%) in older adults who use opioids (OR [95%CI]: 5.50 [1.22–24.76]; *p* = 0.022).

### 3.3. Attitudes

Despite an upward trend, there was no statistically significant change in the mean (SD) prescribing and dispensing attitudes from baseline to post-conference (3.99 [0.62] versus 4.04 [0.56]; *r* = 0.065, *p* = 0.587) (Cronbach’s alpha = 0.728–0.700) (Table 4). Specifically, participants agreed or strongly agreed that they were willing to treat older adults using medications for opioid use disorder (e.g., buprenorphine) at a rate of 70% pre-conference, increasing to 76% post-conference (Table 5). Interestingly, the proportion of respondents willing to treat older adults using non-pharmacological approaches for opioid use disorder (e.g., behavioral therapy) (85.7% at baseline and 88.9% post-conference) exceeded those willing to use pharmacologic approaches. There was a similar trend among participants regarding whether all older adult patients prescribed opioids should be co-prescribed naloxone, with 73.2% of participants agreeing pre-conference and increasing to 83.3% post-conference. Additionally, willingness to treat older adults for pain was higher using non-opioid analgesics versus opioid analgesics at both pre-conference (85.7% vs. 65.2%) and post-conference (79.1% vs. 68.1%).

### 3.4. Perceived Susceptibility

Mean (SD) perceived susceptibility to harm from opioids increased from pre- to post-conference (3.82 [0.63] versus 4.03 [0.63]; *r* = 0.376, *p* = 0.002) (Cronbach’s alpha = 0.680–0.800) (Table 4). In particular, the proportion of respondents who agreed or strongly agreed that older adults are more vulnerable to fatal opioid overdose increased by 11.7% from pre- to post-conference, with a 13.3% increase in those who believed that older adults are less likely to be retained in OUD treatment (Table 5).

### 3.5. Perceived Barriers

Overall, mean (SD) perceived barriers to opioid harm reduction in older adults decreased from pre- to post-conference (2.71 [0.54] versus 2.54 [0.58]; *r* = 0.388, *p* = 0.001) (Cronbach’s alpha = 0.852–0.887) (Table 4). The starkest differences between pre- and post-conference results were seen in the items relating to prescribing/dispensing analgesics or adjuvants for pain to older adults (Table 5). For example, 45.6% of participants agreed or strongly agreed at pre-conference that they did not have enough training to prescribe/dispense opioid analgesics to older adults, with only 22.5% agreeing post-conference. Similarly, participants agreed that they did not have enough training to prescribe/dispense non-opioid analgesics to older adults at a rate of 35.7% pre-conference, with only 14.1% agreeing post-conference. Other major barriers cited by attendees included perceived patient resistance to OUD treatment (up to 46.5%) and lack of reliable transportation (up to 43.6%) among older adult patients.

### 3.6. Intentions

At post-conference, 34.7% of respondents intended to join the Microsoft Teams working group, 12.0% did not intend to join, and 53.3% were unsure. As of 30 July 2025 (3 months post-conference), *n* = 157 attendees (84.0% of all conference attendees) had joined the Teams working group.

## 4. Discussion

There was a small but statistically significant increase in knowledge regarding opioid use and misuse among older adults from pre- to post-conference. This increase indicates the effectiveness of enhancing provider knowledge of OUD in vulnerable populations through interprofessional education. These results align with previously published literature, which suggests that interprofessional education is effective at increasing knowledge [36,37,38]. Of note, healthcare professional knowledge surrounding the risks and consequences of opioid use/misuse in older adults was already high at baseline, indicating current PharmD curricula [36,37] and professional development initiatives [38] adequately address these topics. However, the largest gap in provider knowledge in the current study surrounded OUD treatment, indicating that this is an area for future curricular development.

Prescribing and dispensing attitudes surrounding opioids and MOUD in older adults remained high, with no statistically significant change from pre- to post-conference. Across timepoints, willingness to treat older adults for pain using non-opioid analgesics exceeded provider willingness to utilize opioid analgesics for pain management in this population. This is not unexpected, as it aligns with prior research indicating that prescribers struggle with balancing the risks of opioids in older adults (e.g., sedation, falls) with adequate pain relief [39,40,41]. Interestingly, attendees also viewed non-pharmacologic approaches to treatment of opioid use disorder more favorably than MOUD in this population. While multiple previous studies have examined healthcare provider beliefs regarding MOUD [42,43,44,45], little work to date has focused on comparing provider perceptions surrounding pharmacologic versus non-pharmacologic OUD treatment modalities in older adults. This represents an opportunity for future research to better understand the decision-making schemas and leverage points underlying geriatric OUD management.

Additionally, there was a moderate, statistically significant increase in perceived susceptibility of older adults to opioid-related harm across timepoints, indicating heightened recognition of patient risk [46]. In particular, the altered perceptions in the current study, especially regarding vulnerability to fatal overdoses and challenges surrounding retention in OUD treatment, indicate an enhanced understanding of the nuances involved in the care of older adults who use opioids. This aligns with previous research noting a heightened recognition of risks associated with opioid prescribing among older adult patients after an interprofessional educational initiative for health professions trainees [47]. Given that risk perception has been shown to motivate practice change [48], future studies should investigate whether this change in perceived susceptibility to opioid-related harms translates to changes in provider behavior and implementation of risk-mitigation strategies such as OUD screening, communication, and retention procedures tailored to the older adult population [49,50]. Furthermore, while multiple studies have assessed provider attitude as it relates to their prescriptive authority [51,52], there is limited literature assessing perceived susceptibility to opioid-related harm across vulnerable patient populations and provider types. Therefore, subsequent studies may wish to further investigate how perceived susceptibility to harm from opioids may vary across provider types and settings, providing a foundation for the development of future targeted educational programs for healthcare professionals.

Perceived barriers to opioid harm reduction in older adults moderately decreased from pre- to post-conference. Of note, major barriers pre-conference revolved around healthcare providers’ perceived lack of training in prescribing and/or dispensing of opioid and non-opioid analgesics as well as adjuvants for pain. This aligns with previous literature that found that providers lacked confidence in their ability to treat patients with chronic pain [53]. Future educational programs may wish to focus on content related to prescribing guidelines for opioids as well as non-opioid analgesics and adjuvants for pain. Additionally, perceived older adult resistance to OUD treatment emerged as a commonly cited barrier at both timepoints, consistent with prior research that identified perceived “resistance to change” as a major barrier to treatment among older adults with substance use disorder [54]. Such perceptions may be further shaped by the broader context in which providers operate; current literature suggests that healthcare professionals who frequently work with patients with OUD experience elevated levels of stigma, stress, and burnout compared with their peers [55], potentially influencing how they interpret and respond to patient behaviors across age groups. Future studies may wish to investigate provider perceptions across patient ages, as well as the impact of age-tailored strategies to overcome patient resistance to OUD screening and treatment [56], such as adapting motivational interviewing for potential cognitive limitations or age-specific motivators in older adults [57,58].

Furthermore, about one-third of survey respondents intended to join the Microsoft Teams working group post-conference, while approximately half were undecided. Interestingly, 84% of all attendees had joined the working group at 3 months post-conference. This pattern aligns with literature showing that participation in virtual communities of practice often increases over time as users become more familiar with the platform and perceive value in shared professional learning [59,60]. Future investigation of factors (e.g., knowledge, beliefs, sociodemographics) influencing providers’ intention and ultimate decision to join the Teams working group may provide insight into drivers and leverage points to target in subsequent interventions. Of note, joining the Teams page (accepting the invitation) does not indicate level of engagement in the working group. Future studies may wish to adapt this virtual model of interprofessional collaboration and evaluate level of engagement (e.g., number of posts, active discussions) across disciplines and over time. Prior research demonstrates that participation levels in virtual educational or professional communities frequently vary, with most users acting as passive observers and a smaller subset driving active discussion [61], an important consideration for interpreting engagement metrics in digital interprofessional groups that is beyond the scope of the current study.

### Limitations

The study was limited by a small sample size due to poor retention of participants who completed both pre- and post-conference surveys. Statistical power was still achieved for the pre-post analyses conducted in the current study; however, the sample size was insufficient to evaluate predictors of intention to join the Teams working group via regression analysis. Furthermore, outcomes were assessed at only two timepoints, which restricted the ability to evaluate longer-term changes in knowledge and beliefs. Future work should incorporate multiple follow-up assessments to determine whether gains are sustained over time. Additionally, the sample consisted predominantly of White, female pharmacists, resulting in limited diversity across race/ethnicity, sex, and professional roles, and constraining the generalizability of findings. Similarly, although recruitment targeted a national audience of healthcare providers, the majority of participants were pharmacists and located in Alabama; therefore, findings primarily reflect a regional sample of pharmacists, limiting generalizability across geographic regions and professions. Future studies would benefit from broader recruitment strategies to ensure a more diverse participant pool. In addition, while the HCOP Office of Continuing Education newsletter had an approximate open rate of 45% (2308 opens), the open rate for the COACH listserv was not available. Given this limited tracking of email reach and engagement, as well as subscriber overlap between listservs, a response rate could not be calculated, limiting assessment of potential non-response bias.

Further, social desirability and selection biases remain inherent challenges in survey research; although difficult to eliminate entirely, the anonymous nature of the surveys likely helped attenuate their impact. Similarly, history and maturation biases limit the internal validity and causal conclusions that can be drawn from single-group pretest–posttest studies; future studies may consider utilizing a two-group or delayed intervention design to minimize these biases. In addition, although survey items were designed to be role-inclusive and applicable across professions, wording such as “prescribe/dispense” may have been interpreted differently depending on professionals’ scope of practice, potentially introducing variability in responses. Along the same lines, professionals’ state of practice was not collected, which may have influenced responses if it differed from their state of residence.

## 5. Conclusions

Overall, healthcare professional knowledge of and perceived susceptibility to opioid-related harms among older adults increased while perceived barriers to harm reduction strategies decreased from pre-to-post conference. Opioid and MOUD prescribing and dispensing attitudes remained stable across timepoints. Findings support the potential utility of interprofessional educational interventions to increase healthcare provider knowledge and beliefs regarding opioid harm reduction strategies amongst older adults. Future studies should investigate sustainment of knowledge and beliefs over time and assess whether these gains influence changes in practice behavior and implementation of risk-mitigation strategies.

## Figures and Tables

**Table 1:** Didactic Lectures and Learning Objectives.

Session Title	Learning Objectives	Speaker Credentials
Current Overdose Trends in the United States and in Older Adults	Review current trends in the opioid epidemic and substance use disorders. Discuss the relevance of substance use disorder trends to older adult populations. Examine prescription and illicit potential drugs of abuse.	PharmD, Board Certified Pharmacotherapy Specialist, Board Certified Geriatric Pharmacist
Opioid Prescribing in the Elderly	Review CDC prescribing guidelines for opioids with special attention to older adults. Discuss options for treating pain in the population of older adults. Utilize case studies to analyze practical applications of utilizing opioids for pain management in older adults with comorbid conditions.	PharmD, COACH Director, Board Certified Pharmacotherapy Specialist, Credentialed in Pain Management by the American Academy of Pain Management, Certified Pain Educator
Introduction to Medications for Treatment of Opioid Use Disorder	Define opioid use disorder (OUD). Describe medications to treat OUD. Examine myths related to OUD. Discuss the benefits and risks specific to the older adult population related to OUD.	PharmD, Board Certification in Psychiatric Pharmacotherapy
Substance Use Resources for Older Populations	Identify resources available for those with SUD. Explain how individuals gain access to treatment resources. Describe challenges and barriers for individuals with SUD to receive resources.	MS, Advanced Alcohol and Drug Counselor (AADC)
Managing Opioid Overdose and Naloxone	Describe how to manage an opioid overdose. Apply opioid overdose management prevention strategies to older adult patients. Demonstrate how to administer naloxone in the case of an opioid overdose.	PharmD, PhD, Medication Therapy Management (MTM) Certified
Prescription Drug Monitoring Program (PDMP)	Discuss the utilization of Prescriber and Patient reports available in the Prescription Drug Monitoring Program (PDMP). Review “Best Practices” for utilizing the PDMP to assess a patient’s risk of misuse or overdose. Evaluate a patient case using data housed in the PDMP.	PharmD, Board Certified Ambulatory Care Pharmacist
Drugs of Abuse and Fentanyl Test Strips	List the most common substances used by older adults. Describe the role of fentanyl in the recent increase in overdose deaths. Explain how to use fentanyl test strips. Identify some limitations of fentanyl test strips.	MD, Board Certified in Adult Psychiatry and Addiction Medicine
Medication Labeling Best Practices Among Older Adults	Identify common medication errors in older adults. Determine medication labeling best practices for older adults.	PharmD, Board Certified Ambulatory Care Pharmacist
Enhancing Patient Care Through Collaboration	Explore participant base knowledge of conference related subject matter. Orient participants to networking platform/resources to encourage participants to collaborate post conference. Examine the effects of an interdisciplinary approach to patient care.	PhD, COACH Post-Doctoral Fellow

**Table 2:** Participant Demographics (*n* = 75).

Questions	*n* (%) ^a^
Profession	
Behavioral or mental health specialist	2 (2.7)
Nurse	6 (8.0)
Nurse practitioner	2 (2.7)
Pharmacist	51 (68.0)
Pharmacy technician	3 (4.0)
Physician	2 (2.7)
Social worker	6 (8.0)
Other	3 (4.0)
Sex	
Female	56 (74.7)
Male	19 (25.3)
Race or ethnicity ^b^	
American Indian or Alaska Native	0 (0)
Asian	1 (1.3)
Black or African American	9 (12.0)
Hispanic or Latino(a)	0 (0)
Native Hawaiian or other Pacific Islander	0 (0)
White	65 (86.7)
Other	2 (2.6)
State of residence	
Alabama	58 (78.4)
Georgia	4 (5.4)
Hawaii	1 (1.4)
Kentucky	2 (2.7)
Louisiana	1 (1.4)
Mississippi	3 (4.1)
North Carolina	1 (1.4)
Pennsylvania	1 (1.4)
Tennessee	3 (4.1)
Urbanicity	
Rural	11 (15.1)
Urban	62 (84.9)
**Questions**	**Mean (SD)** **Median (IQR)**
Age, years	49.88 (13.63) 48.00 (38.50, 60.00)

^a^ Frequencies and percentages may differ due to item non-response. ^b^ Participants were instructed to select all that apply.

**Table 3:** Healthcare Provider Knowledge Regarding Opioid Use and Misuse in Older Adults (*n* = 75).

Measure	Pre	Post		
	Mean (SD) Median (IQR)		Effect Size ^a^	*p*-Value
Overall Knowledge Score, % Correct	83.73 (19.92) 80.00 (80.00, 100.00)	90.67 (12.45) 100.00 (80.00, 100.00)	0.295	0.011
**Items**	***n* (%)**		**Effect Size ^b^**	***p*-Value**
Only 20% of people receive needed treatment for opioid use disorder (OUD) True * False Unsure	48 (64.0) 16 (21.3) 11 (14.7)	58 (78.4) 15 (20.3) 1 (1.3)	2.38 (1.04–5.43)	0.054
Changes in opioid pharmacokinetics with age lead to increased likelihood of negative side effects from opioids True * False Unsure	70 (93.3) 4 (5.3) 1 (1.3)	73 (97.3) 1 (1.3) 1 (1.3)	2.50 (0.48–12.88)	0.453
Older adults and elderly (≥65) who use opioids are at increased risk of the following. Please check all that apply. Respiratory depression * Sedation * Overdose * None of the above Unsure	71 (94.7) 69 (92) 66 (88) 0 (0) 1 (1.3)	75 (100) 73 (97.3) 72 (96.0) 0 (0) 0 (0)	5.50 (1.22–24.76)	0.022
About 10% of older adults progress to chronic (>3 months) opioid use after surgery True * False Unsure	61 (81.3) 5 (6.7) 9 (12.0)	62 (82.7) 9 (12.0) 4 (5.3)	1.11 (0.45–2.73)	1.000
The odds of developing OUD are exacerbated in older adults experiencing social isolation True * False Unsure	72 (96.0) 0 (0) 3 (4.0)	74 (98.7) 1 (1.3) 0 (0)	3.00 (0.31–28.80)	0.625

* Correct response. ^a^ Effect size: *r* = |Z|/√N. ^b^ Effect size: McNemar Odds Ratio (95% Confidence Interval) for correct versus incorrect response.

**Table 4:** Changes in Overall Attitudes, Perceived Susceptibility, and Perceived Barriers Scale Scores from Pre- to Post-Conference (*n* = 75).

Measures	Time	Cronbach’s Alpha	Mean (SD) Median (IQR)	Effect Size ^a^	*p*-Value
Prescribing and Dispensing Attitudes Scale	Pre	0.728	3.99 (0.62) 4.00 (3.60, 4.40)	0.065	0.587
	Post	0.700	4.04 (0.56) 4.00 (3.80, 4.40)		
Perceived Susceptibility to Harm Scale	Pre	0.680	3.82 (0.63) 4.00 (3.50, 4.25)	0.376	0.002
	Post	0.800	4.03 (0.63) 4.00 (3.50, 4.50)		
Perceived Barriers Scale	Pre	0.852	2.71 (0.54) 2.71 (2.35, 3.06)	0.388	0.001
	Post	0.887	2.54 (0.58) 2.47 (2.18, 2.94)		

^a^ Effect size: *r* = |Z|/√N.

**Table 5:** Healthcare Provider Beliefs Regarding Opioid Use/Misuse in Older Adults (*n* = 75).

Items	Time	*n* (%)				
		1	2	3	4	5
Prescribing and Dispensing Attitudes Surrounding Opioids and MOUD						
I am willing to treat older adults for pain using opioid analgesics	Pre	2 (2.9)	3 (4.3)	19 (27.5)	35 (50.7)	10 (14.5)
	Post	1 (1.4)	3 (4.2)	19 (26.4)	36 (50.0)	13 (18.1)
I am willing to treat older adults for pain using non-opioid analgesics	Pre	2 (2.9)	0 (0)	8 (11.4)	32 (45.7)	28 (40.0)
	Post	1 (1.4)	0 (0)	14 (19.4)	34 (47.2)	23 (31.9)
I am willing to treat older adults using medications for opioid use disorder (e.g., buprenorphine)	Pre	2 (2.9)	2 (2.9)	17 (24.3)	34 (48.6)	15 (21.4)
	Post	1 (1.4)	1 (1.4)	15 (20.8)	39 (54.2)	16 (22.2)
I am willing to treat older adults using non-pharmacological approaches for opioid use disorder (e.g., behavioral therapy)	Pre	1 (1.4)	0 (0)	9 (12.9)	33 (47.1)	27 (38.6)
	Post	2 (2.8)	0 (0)	6 (8.3)	34 (47.2)	30 (41.7)
All older adult patients prescribed opioids should be co-prescribed naloxone	Pre	3 (4.2)	2 (2.8)	14 (19.7)	29 (40.8)	23 (32.4)
	Post	0 (0)	5 (6.9)	7 (9.7)	36 (50.0)	24 (33.3)
Perceived Susceptibility to Harm from Opioids						
Older adults are more vulnerable to fatal opioid overdose	Pre	1 (1.4)	3 (4.3)	12 (17.1)	31 (44.3)	23 (32.9)
	Post	0 (0)	1 (1.4)	7 (9.7)	36 (50.0)	28 (38.9)
Older adults are more likely to experience opioid use disorder	Pre	2 (2.8)	8 (11.3)	22 (31.0)	28 (39.4)	11 (15.5)
	Post	0 (0)	9 (12.3)	18 (24.7)	32 (43.8)	14 (19.2)
Older adults are less likely to initiate treatment for OUD	Pre	1 (1.4)	2 (2.8)	9 (12.7)	41 (57.7)	18 (25.4)
	Post	0 (0)	3 (4.1)	3 (4.1)	42 (57.5)	25 (34.2)
Older adults are less likely to be retained in OUD treatment	Pre	1 (1.4)	5 (7.0)	18 (25.4)	40 (56.3)	7 (9.9)
	Post	0 (0)	6 (8.2)	9 (12.3)	41 (56.2)	17 (23.3)
Perceived Barriers to Opioid Harm Reduction						
I do not have enough training to prescribe/dispense opioid analgesics to older adults	Pre	4 (5.9)	17 (25.0)	16 (23.5)	21 (30.9)	10 (14.7)
	Post	9 (12.7)	27 (38.0)	19 (26.8)	14 (19.7)	2 (2.8)
I do not have enough training to prescribe/dispense non-opioid analgesics to older adults	Pre	7 (10.0)	21 (30.0)	17 (24.3)	18 (25.7)	7 (10.0)
	Post	15 (21.1)	33 (46.5)	13 (18.3)	8 (11.3)	2 (2.8)
I do not have enough training to prescribe/dispense adjuvants for pain (e.g., gabapentin) to older adults	Pre	6 (8.6)	23 (32.9)	21 (30.0)	13 (18.6)	7 (10.0)
	Post	11 (15.5)	30 (42.3)	19 (26.8)	9 (12.7)	2 (2.8)
There is no demand in my practice for naloxone among older adults	Pre	12 (17.1)	24 (34.3)	19 (27.1)	12 (17.1)	3 (4.3)
	Post	18 (25.4)	20 (28.2)	23 (32.4)	7 (9.9)	3 (4.2)
There is no demand in my practice for medications for opioid use disorder (e.g., buprenorphine) among older adults	Pre	13 (18.6)	23 (32.9)	18 (25.7)	12 (17.1)	4 (5.7)
	Post	15 (21.1)	27 (38.0)	20 (28.2)	6 (8.5)	3 (4.2)
There is no demand in my practice for non-pharmacologic treatments for opioid use disorder (e.g., behavioral therapy) among older adults	Pre	13 (18.6)	22 (31.4)	19 (27.1)	12 (17.1)	4 (5.7)
	Post	19 (26.8)	25 (35.2)	20 (28.2)	5 (7.0)	2 (2.8)
It is too difficult to treat older adults for opioid use disorder	Pre	10 (14.3)	26 (37.1)	25 (35.7)	5 (7.1)	4 (5.7)
	Post	15 (21.1)	33 (46.5)	15 (21.1)	7 (9.9)	1 (1.4)
Older patients do not show up to appointments	Pre	4 (5.7)	32 (45.7)	29 (41.4)	5 (7.1)	0 (0)
	Post	11 (15.5)	30 (42.3)	25 (35.2)	3 (4.2)	2 (2.8)
Older adult patients are resistant to treatment for opioid use disorder	Pre	2 (2.9)	20 (29.0)	25 (36.2)	21 (30.4)	1 (1.4)
	Post	3 (4.2)	18 (25.4)	17 (23.9)	27 (38.0)	6 (8.5)
Older adult patients are resistant to receiving naloxone	Pre	1 (1.4)	22 (31.4)	24 (34.3)	20 (28.6)	3 (4.3)
	Post	2 (2.8)	23 (32.4)	18 (25.4)	26 (36.6)	2 (2.8)
I am hesitant to talk to my older adult patients about harm reduction for fear that they will react negatively	Pre	11 (15.7)	34 (48.6)	18 (25.7)	6 (8.6)	1 (1.4)
	Post	13 (18.3)	29 (40.8)	18 (25.4)	10 (14.1)	1 (1.4)
It takes too much time to address harm reduction measures with older adult patients	Pre	14 (20.3)	32 (46.4)	19 (27.5)	3 (4.3)	1 (1.4)
	Post	16 (22.9)	38 (54.3)	11 (15.7)	4 (5.7)	1 (1.4)
There is not enough staff at my practice site to address harm reduction measures for older adult patients	Pre	3 (4.3)	21 (30.4)	27 (39.1)	15 (21.7)	3 (4.3)
	Post	5 (7.0)	21 (29.6)	29 (40.8)	14 (19.7)	2 (2.8)
Managers/supervisors at my practice site are not supportive of harm reduction for older adult patients	Pre	10 (14.3)	29 (41.4)	22 (31.4)	8 (11.4)	1 (1.4)
	Post	13 (18.3)	34 (47.9)	18 (25.4)	3 (4.2)	3 (4.2)
Co-workers at my practice site are not supportive of harm reduction for older adult patients	Pre	10 (14.3)	38 (54.3)	18 (25.7)	3 (4.3)	1 (1.4)
	Post	12 (16.9)	37 (52.1)	15 (21.1)	4 (5.6)	3 (4.2)
There are not enough resources at my practice site for harm reduction in older adult patients	Pre	6 (8.6)	24 (34.3)	25 (35.7)	15 (21.4)	0 (0)
	Post	8 (11.3)	24 (33.8)	21 (29.6)	17 (23.9)	1 (1.4)
Older adult patients have difficulty finding reliable transportation to my practice site	Pre	1 (1.4)	6 (8.7)	32 (46.4)	26 (37.7)	4 (5.8)
	Post	0 (0)	10 (14.1)	30 (42.3)	27 (38.0)	4 (5.6)

1 = strongly disagree, 2 = disagree, 3 = neutral, 4 = agree, 5 = strongly agree.

## Data Availability

The data presented in this study are available on request from the corresponding author due to restrictions within the Institutional Review Board protocol.

## Supplementary Materials

The following supporting information can be downloaded at: https://www.mdpi.com/article/10.3390/pharmacy14030086/s1, File S1: Survey Instruments; and File S2: Transparent Reporting of Evaluations with Nonrandomized Designs (TREND) checklist.

## Abbreviations

The following abbreviations are used in this manuscript:

CDC	Centers for Disease Control and Prevention
HBM	Health Belief Model
MOUD	Medications for opioid use disorder
OUD	Opioid use disorder
SD	Standard deviation
TPB	Theory of Planned Behavior
