# Supplementary material for: Impact of an Interdisciplinary Educational Intervention on Healthcare Provider Knowledge and Beliefs Regarding Opioid Harm Reduction in Older Adults: A Pre-Post Survey Study
Source: Pharmacy (Basel). 2026 Jun 16;14(3):86. doi: 10.3390/pharmacy14030086 (PMC13306577; doi:10.3390/pharmacy14030086)
Supplement: Supplementary file 1 [file pharmacy-14-00086-s001.zip › Supplemental File S1_survey instruments.pdf]

2025 COACH CONFERENCE:
DISPARITIES IN OPIOID HARM REDUCTION AMONG OLDER ADULTS
Pre-Survey

I. PERSONAL SURVEY CODE

1. Please develop your own PERSONAL SURVEY CODE by listing the **FIRST 2 letters of your mother's maiden name** plus the **LAST 3 digits of your phone number** below (e.g., AB123). This personal survey code will maintain the anonymity of your data, while allowing us to match your pre-conference and post-conference survey responses together for research purposes.

PERSONAL SURVEY CODE

FIRST 2 letters of mother's maiden name + LAST 3 digits of your phone number (e.g., AB123): ________________

II. INFORMATION ABOUT YOU

2. Please indicate your profession:
☐ Dentist ☐ Dental hygienist ☐ Nurse (RN, LPN) ☐ Nurse practitioner
☐ Pharmacist ☐ Pharmacy technician ☐ Physician ☐ Physician assistant (PA)
☐ EMT ☐ Social worker ☐ Behavioral or mental health specialist
☐ Other. Please specify: ___________________

3. What is your sex?
☐ Male ☐ Female ☐ Non-binary

4. With which race or ethnicity do you most closely identify? Please check all that apply.
☐ White ☐ Black or African American ☐ American Indian or Alaska Native ☐ Asian ☐ Latino(a)
☐ Native Hawaiian or Other Pacific Islander ☐ Other. Please specify: ___________________

5. Please enter your age in years: __________________

6. Please indicate the State and Zip Code in which you reside:
State: ___________________ [dropdown list]
Zip Code: ________________ [text entry]

III. KNOWLEDGE REGARDING OPIOID HARM REDUCTION IN OLDER ADULTS

7. Only 20% of people receive needed treatment for opioid use disorder (OUD).
☐ True ☐ False ☐ Unsure

8. Changes in opioid pharmacokinetics with age lead to increased likelihood of negative side effects from opioids.
☐ True ☐ False ☐ Unsure

9. Older adults and elderly (≥65) who use opioids are increased risk of the following. Please check all that apply.
☐ Respiratory depression ☐ Sedation ☐ Overdose ☐ None of the above ☐ Unsure

10. About 10% of older adults progress to chronic (> 3 months) opioid use after surgery.
☐ True ☐ False ☐ Unsure

11. The odds of developing OUD are exacerbated in older adults experiencing social isolation.
☐ True ☐ False ☐ Unsure

IV. ATTITUDES REGARDING OPIOID HARM REDUCTION AMONG OLDER ADULTS

12. On a scale of 1 to 5, please indicate your level of agreement or disagreement with the following statements regarding your attitudes about opioid harm reduction strategies among older adults, where 1=strongly disagree and 5=strongly agree.

Questions	Strongly Disagree	Disagree	Neutral	Agree	Strongly Agree
I am willing to treat older adults for pain using opioid analgesics	☐	☐	☐	☐	☐

I am willing to treat older adults for pain using non-opioid analgesics	☐	☐	☐	☐	☐
I am willing to treat older adults using medications for opioid use disorder (e.g., buprenorphine)	☐	☐	☐	☐	☐
I am willing to treat older adults using non-pharmacological approaches for opioid use disorder (e.g., behavioral therapy)	☐	☐	☐	☐	☐
All older adult patients prescribed opioids should be co-prescribed naloxone	☐	☐	☐	☐	☐
Older adults are more vulnerable to fatal opioid overdose	☐	☐	☐	☐	☐
Older adults are more likely to experience opioid use disorder (OUD)	☐	☐	☐	☐	☐
Older adults are less likely to initiate treatment for OUD	☐	☐	☐	☐	☐
Older adults are less likely to be retained in OUD treatment	☐	☐	☐	☐	☐

V. PERCEIVED BARRIERS REGARDING OPIOID HARM REDUCTION AMONG OLDER ADULTS

13. On a scale of 1 to 5, please indicate your level of agreement or disagreement with the following statements regarding factors that make opioid harm reduction more difficult among older adults, where 1=strongly disagree and 5=strongly agree.

**Questions**	**Strongly Disagree**	**Disagree**	**Neutral**	**Agree**	**Strongly Agree**
I do not have enough training to prescribe/dispense opioid analgesics to older adults	☐	☐	☐	☐	☐
I do not have enough training to prescribe/dispense non-opioid analgesics to older adults	☐	☐	☐	☐	☐
I do not have enough training to prescribe/dispense adjuvants for pain (e.g., gabapentin) to older adults	☐	☐	☐	☐	☐
There is no demand in my practice for naloxone among older adults	☐	☐	☐	☐	☐
There is no demand in my practice for medications for opioid use disorder (e.g., buprenorphine) among older adults	☐	☐	☐	☐	☐
There is no demand in my practice for non-pharmacologic treatments for opioid use disorder (e.g., behavioral therapy) among older adults	☐	☐	☐	☐	☐
It is too difficult to treat older adults for opioid use disorder	☐	☐	☐	☐	☐
Older adult patients do not show up to appointments	☐	☐	☐	☐	☐
Older adult patients are resistant to treatment for opioid use disorder	☐	☐	☐	☐	☐
Older adult patients are resistant to receiving naloxone	☐	☐	☐	☐	☐
I am hesitant to talk to my older adult patients about harm reduction for fear that they will react negatively	☐	☐	☐	☐	☐
It takes too much time to address harm reduction measures with older adult patients	☐	☐	☐	☐	☐
There is not enough staff at my practice site to address harm reduction measures for older adult patients	☐	☐	☐	☐	☐
Managers/supervisors at my practice site are not supportive of harm reduction for older adult patients	☐	☐	☐	☐	☐
Co-workers at my practice site are not supportive of harm reduction for older adult patients	☐	☐	☐	☐	☐
There are not enough resources at my practice site for harm reduction for older adult patients	☐	☐	☐	☐	☐
Older adult patients have difficulty finding reliable transportation to my practice site	☐	☐	☐	☐	☐

2025 COACH CONFERENCE:
DISPARITIES IN OPIOID HARM REDUCTION AMONG OLDER ADULTS
Post-Survey

I. PERSONAL SURVEY CODE

1. Please provide your PERSONAL SURVEY CODE from the pre-survey by listing the **FIRST 2 letters of your mother's maiden name** plus the **LAST 3 digits of your phone number** below (e.g., AB123). This personal survey code will maintain the anonymity of your data, while allowing us to match your pre-conference and post-conference survey responses together for research purposes.

PERSONAL SURVEY CODE

FIRST 2 letters of mother's maiden name + LAST 3 digits of your phone number(e.g., AB123): ________________

II. KNOWLEDGE REGARDING OPIOID HARM REDUCTION IN OLDER ADULTS

2. Only 20% of people receive needed treatment for opioid use disorder (OUD).
   ☐ True ☐ False ☐ Unsure

3. Changes in opioid pharmacokinetics with age lead to increased likelihood of negative side effects from opioids.
   ☐ True ☐ False ☐ Unsure

4. Older adults and elderly (≥65) who use opioids are increased risk of the following. Please check all that apply.
   ☐ Respiratory depression ☐ Sedation ☐ Overdose ☐ None of the above ☐ Unsure

5. About 10% of older adults progress to chronic (> 3 months) opioid use after surgery.
   ☐ True ☐ False ☐ Unsure

6. The odds of developing OUD are exacerbated in older adults experiencing social isolation.
   ☐ True ☐ False ☐ Unsure

III. ATTITUDES REGARDING OPIOID HARM REDUCTION AMONG OLDER ADULTS

7. On a scale of 1 to 5, please indicate your level of agreement or disagreement with the following statements regarding your attitudes about opioid harm reduction strategies among older adults, where 1=strongly disagree and 5=strongly agree.

Questions	Strongly Disagree	Disagree	Neutral	Agree	Strongly Agree
I am willing to treat older adults for pain using opioid analgesics	☐	☐	☐	☐	☐
I am willing to treat older adults for pain using non-opioid analgesics	☐	☐	☐	☐	☐
I am willing to treat older adults using medications for opioid use disorder (e.g., buprenorphine)	☐	☐	☐	☐	☐
I am willing to treat older adults using non-pharmacological approaches for opioid use disorder (e.g., behavioral therapy)	☐	☐	☐	☐	☐
All older adult patients prescribed opioids should be co-prescribed naloxone	☐	☐	☐	☐	☐
Older adults are more vulnerable to fatal opioid overdose	☐	☐	☐	☐	☐
Older adults are more likely to experience opioid use disorder (OUD)	☐	☐	☐	☐	☐
Older adults are less likely to initiate treatment for OUD	☐	☐	☐	☐	☐
Older adults are less likely to be retained in OUD treatment	☐	☐	☐	☐	☐

IV. PERCEIVED BARRIERS REGARDING OPIOID HARM REDUCTION AMONG OLDER ADULTS

8. On a scale of 1 to 5, please indicate your level of agreement or disagreement with the following statements regarding factors that make opioid harm reduction more difficult among older adults, where 1=strongly disagree and 5=strongly agree.

Questions	Strongly Disagree	Disagree	Neutral	Agree	Strongly Agree
I do not have enough training to prescribe/dispense opioid analgesics to older adults	☐	☐	☐	☐	☐
I do not have enough training to prescribe/dispense non-opioid analgesics to older adults	☐	☐	☐	☐	☐
I do not have enough training to prescribe/dispense adjuvants for pain (e.g., gabapentin) to older adults	☐	☐	☐	☐	☐
There is no patient demand in my practice for naloxone among older adults	☐	☐	☐	☐	☐
There is no demand in my practice for medications for opioid use disorder (e.g., buprenorphine) among older adults	☐	☐	☐	☐	☐
There is no demand in my practice for non-pharmacologic treatments for OUD (e.g., behavioral therapy) among older adults	☐	☐	☐	☐	☐
It is too difficult to treat older adults for opioid use disorder	☐	☐	☐	☐	☐
Older adult patients do not show up to appointments	☐	☐	☐	☐	☐
Older adult patients are resistant to treatment for opioid use disorder	☐	☐	☐	☐	☐
Older adult patients are resistant to receiving naloxone	☐	☐	☐	☐	☐
I am hesitant to talk to my older adult patients about harm reduction for fear that they will react negatively	☐	☐	☐	☐	☐
It takes too much time to address harm reduction measures with older adult patients	☐	☐	☐	☐	☐
There is not enough staff at my practice site to address harm reduction measures for older adult patients	☐	☐	☐	☐	☐
Managers/supervisors at my practice site are not supportive of harm reduction for older adult patients	☐	☐	☐	☐	☐
Co-workers at my practice site are not supportive of harm reduction for older adult patients	☐	☐	☐	☐	☐
There are not enough resources at my practice site for harm reduction for older adult patients	☐	☐	☐	☐	☐
Older adult patients have difficulty finding reliable transportation to my practice site	☐	☐	☐	☐	☐

9. Do you intend to join the Microsoft Teams COACH Annual Conference working group?
   ☐ Yes ☐ No ☐ Unsure

10. What strategies do you recommend to form an interdisciplinary network of professionals for future opioid research, education, and outreach collaborations focused on older adults?

