# Supplementary material for: Impact of an Interdisciplinary Educational Intervention on Healthcare Provider Knowledge and Beliefs Regarding Opioid Harm Reduction in Older Adults: A Pre-Post Survey Study
Source: Pharmacy (Basel). 2026 Jun 16;14(3):86. doi: 10.3390/pharmacy14030086 (PMC13306577; doi:10.3390/pharmacy14030086)
Supplement: Supplementary file 1 [file pharmacy-14-00086-s001.zip › Supplemental File S2_TREND checklist.pdf]

Supplementary File S2. TREND Reporting Checklist

Section	Item No.	TREND Item Description	Reported?	Location in Manuscript
**Title and Abstract**				
Title and Abstract	1	Information on how units were allocated to interventions, information on the target population or study sample, and a structured abstract.	Yes	Title; Abstract; Methods 2.1
**Introduction**				
Background	2	Scientific background and explanation of rationale for the study.	Yes	Introduction
**Methods**				
Participants	3	Eligibility criteria for participants, method of recruitment, recruitment setting, and settings/location where data were collected.	Yes	Methods 2.2; 2.5
Intervention	4	Details of the intervention intended for each study condition and how and when it was administered, including content,	Yes	Methods 2.4; Table 1

		delivery method, unit of delivery, deliverer, setting, exposure quantity/duration, time span, and activities to increase adherence.		
Objectives	5	Specific objectives and hypotheses.	Yes	Introduction (final paragraph)
Outcomes	6	Clearly defined primary and secondary outcome measures, data collection methods, and information on validity and reliability of instruments.	Yes	Methods 2.5–2.6; Supplementary File S1
Sample Size	7	How sample size was determined and any interim analyses or stopping rules, if applicable.	Yes	Methods 2.3
Assignment Method	8	Unit of assignment, method used to assign units to study conditions, and methods used to minimize bias due to non-randomization.	Yes	Methods 2.1; 2.5
Blinding (masking)	9	Whether participants, intervention administrators, and outcome assessors were	Not applicable	Educational intervention

		blinded to study condition assignment.		
Unit of Analysis	10	Description of the unit of analysis and, if it differs from the unit of assignment, methods used to account for this.	Yes	Methods 2.6
Statistical Methods	11	Statistical methods used to compare intervention effects and assess change over time.	Yes	Methods 2.6
**Results**				
Participant Flow	12	Flow of participants through each stage of the study (enrollment, follow-up, analysis).	Yes	Results 3.1
Recruitment	13	Dates defining the periods of recruitment and follow-up.	Yes	Methods 2.1; Results 3.1
Baseline Data	14	Baseline demographic and clinical characteristics of participants.	Yes	Results 3.1; Table 2
Baseline Equivalence	15	Baseline equivalence of groups and statistical methods used to control for baseline	Not applicable	Single-group design

		differences, if applicable.		
Numbers Analyzed	16	Number of participants included in each analysis.	Yes	Results 3.1–3.6
Outcomes and Estimation	17	Results for each primary and secondary outcome, including effect sizes and precision estimates.	Yes	Results 3.2–3.6; Tables 3–4
Ancillary Analyses	18	Results of subgroup or exploratory analyses, if applicable.	Not applicable	No subgroup analyses conducted
Adverse Events	19	Adverse events or unintended effects in each study condition.	Not applicable	No adverse events reported
**Discussion**				
Interpretation	20	Interpretation of the results, considering study hypotheses, sources of bias, and imprecision.	Yes	Discussion
Generalizability	21	Discussion of the generalizability (external validity) of the study findings.	Yes	Discussion; Limitations
Overall Evidence	22	General interpretation of the results in the context of current	Yes	Discussion; Conclusions

evidence and
implications for
practice or policy.

The initial draft of this TREND reporting checklist was created using Microsoft 365 Copilot as an AI-assisted drafting tool. The checklist was then thoroughly reviewed by the authors, cross-checked against the original 22-item TREND Statement, and revised as needed to ensure accuracy and correct application to the study design. The final checklist reflects the authors' independent assessment.
